# A rare case of Crigler–Najjar syndrome type 2: A case report and literature review

**DOI:** 10.1002/ccr3.8176

**Published:** 2023-11-13

**Authors:** Divas Rijal, Prabhat Rijal, Shyam Murti Bohare, Ashish Sanjay Chaudhari, Mandip Dhungel, Mayank Agarwal, Pramish Bhatta, Tulsi Ram Dhakal, Anjali Bishwokarma, Pooja Kafle

**Affiliations:** ^1^ Department of Critical Care medicine Tribhuvan University Teaching Hospital, Maharajgunj Medical Campus Kathmandu Nepal; ^2^ Department of Internal Medicine All India Institute of Medical Sciences Rishikesh Uttarakhand India; ^3^ Nepalgunj Medical College Nepalgunj Nepal; ^4^ Tribhuvan University Teaching Hospital, Maharajgunj Medical Campus Kathmandu Nepal

**Keywords:** clinical isolated jaundice, Crigler–Najjar syndrome type 2, indirect hyperbilirubinemia, phenobarbitone, UGT1A1 deficiency, unconjugated hyperbilirubinemia

## Abstract

**Key Clinical Message:**

Crigler–Najjar syndrome type 2 should be suspected in any young patient presenting with isolated indirect hyperbilirubinemia where all other common etiologies have been excluded. It is a relatively benign condition that responds to phenobarbitone.

**Abstract:**

Crigler–Najjar syndrome (CNS) type 2 is an inborn cause of isolated indirect hyperbilirubinemia characterized by a partial deficiency of the enzyme uridine 5′‐diphosphate‐glucuronosyltransferase (UGT) responsible for bilirubin conjugation. Typically, this condition is diagnosed based on clinical manifestations, supplemented by enzyme analysis if feasible, and exhibits a significant response to phenobarbitone, known for its enzyme‐inducing properties. In this case, we present a young male patient who had experienced recurrent isolated indirect hyperbilirubinemia since early childhood, with negative results in the hemolytic workup. The patient exhibited a UGT1A1 gene defect and demonstrated a highly favorable response to phenobarbitone treatment. The purpose of this report is to raise awareness among physicians about this benign condition and underscore the importance of avoiding unnecessary investigations.

## Graphical abstract

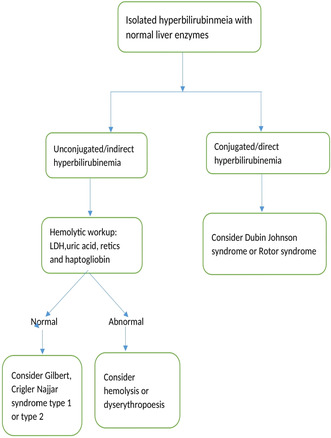

## 1 INTRODUCTION

Crigler–Najjar syndrome is an autosomal recessive inherited disorder characterized by a defect in the activity of the enzyme UDP‐glucuronosyltransferase, required for the glucuronidation of unconjugated bilirubin in the liver.^1^ Based on the activity of the enzyme UDP‐glucuronosyltransferase, Crigler–Najjar syndrome can be divided into two types: type 1 and type 2 with absent or reduced activity of the same enzyme, respectively.^2^ The prevalence of Crigler–Najjar syndrome ranges from 0.6 to 1 case per million live births.^3^ The concentration of unconjugated bilirubin is much higher in type 1 CNS compared to type 2 resulting in the deposition of free bilirubin in the brain manifesting as BIND (Bilirubin‐Induced Neurologic Dysfunction), which includes a spectrum of neurologic abnormalities ranging from subtle findings to kernicterus with permanent neurological deficits.^4^, ^5^, ^6^ CNS type 2 can manifest with episodes of jaundice often triggered by fasting, an intercurrent illness, or hyperbilirubinemia as an incidental lab finding. In the case report below, we present a case summary of a young male who presented to our institute with recurrent episodes of jaundice since the early childhood. He had been troubled with persistent jaundice for the past 6 months accompanied by episodes of non‐projectile vomiting. On a detailed workup of the patient, he was found to have unconjugated hyperbilirubinemia with normal liver enzymes, a negative hemolytic panel, and no evidence of hepatosplenomegaly. An analysis for UGT1A1 mutations demonstrated the partial deficiency of the enzyme activity and was hence diagnosed as Crigler–Najjar syndrome type 2. The patient was then subsequently managed with phenobarbitone therapy, which showed a drastic reduction in serum bilirubin levels. With the case report below, we aim to sensitize clinicians regarding the rare possibility of CNS as a cause of isolated unconjugated hyperbilirubinemia.

## 2 CASE REPORT

A 21‐year‐old male with no history of alcohol, tobacco, or drug abuse presented to our center with a concerning medical background. Since early childhood, he had been plagued by recurrent episodes of jaundice, which prompted him to seek our expertise. The persistent jaundice had been troubling him for the past 6 months, accompanied by occasional bouts of non‐projectile vomiting. His birth history was uneventful, devoid of any complications such as neonatal jaundice or the need for blood transfusions. He achieved the developmental milestones expected for his age, giving no cause for concern. However, when he turned 5 years old, his parents noticed a peculiar yellowish discoloration in his eyes for the first time, which was not associated with fever, itching, abdominal pain, or clay‐colored stools, but they did observe high‐colored urine. Determined to find a remedy, the patient was exposed to various forms of alternative and complementary medicines, although the exact details of these treatments remain undocumented. Regrettably, none of these interventions provided a complete cure for his condition. The patient's parents recollected that his highest recorded serum bilirubin level was 12 mg/dL. Upon our examination, we observed evident jaundice without organomegaly. Furthermore, other clinical evaluations yielded normal results, offering no immediate explanation for his persistent jaundice. Routine investigations, however, revealed the presence of indirect hyperbilirubinemia, accompanied by normal liver enzyme levels. (Table 1) Ultrasonography of the abdomen revealed normal findings. We then proceeded with a comprehensive hemolytic workup, but all the tests came back negative. On the basis of disease onset, course, and the presence of unconjugated hyperbilirubinemia, we tentatively diagnosed the patient with nonhemolytic unconjugated hyperbilirubinemia. With the suspicion of congenital indirect bilirubinemia syndromes, we embarked on an analysis for UGT1A1 mutations, which came positive, indicating the presence of a partial enzyme deficiency. Hence, we confidently arrived at the definitive diagnosis of Crigler–Najjar Syndrome Type 2, an uncommon yet impactful condition. To manage the patient's symptoms and potentially improve his quality of life, we initiated oral phenobarbitone at a dosage of 5 mg/kg. Remarkably, within just 2 weeks of starting the treatment, we observed a significant reduction in the patient's serum bilirubin levels proving itself to be the best therapeutic option.

**TABLE 1:** Investigations.

Investigations	At presentation	After phenobarbitone therapy	Normal Range
TLC (/mm^3^)	8200		4000–11,000/mm^3^
DLC (neutrophils/lymphocytes %)	60/33		Up to 60/40
Hb (g/dL)	13.6		13–16 g/dL
Total bilirubin (mg/dL)	7.5	4.2	0.1–1.0 mg/dL
Direct bilirubin (mg/dL)	0.6	0.5	0–0.3 mg/dL
SGOT (U/L)	31	34	12–38 U/L
SGPT (U/L)	35	40	10–40 U/L
ALP (U/L)	116	108	25–100 U/L
GGT (U/L)	30	26	5–40 U/L
Serum protein (g/dL)	6.6	6.4	6–7.8 g/dL
Serum albumin (g/dL)	4.6	4.6	3.5–5.5 g/dL
Serum globulin (g/dL)	2.8	3	2.3–3.5 g/dL
LDH (U/L)	180		140–280 U/L
Reticulocyte count (%)	2		0.5%–2%
Blood urea (mg/dL)	36		7–18 mg/dL
Serum creatinine (mg/dL)	0.8		0.6–1.2 mg/dL
Urine routine examinatin	Normal		
Viral markers (HIV/HBV/HCV)	Nonreactive		
Special Investigations			
Ultrasonography Abdomen and Pelvis: No abnormality detected Peripheral smear: No abnormality detected Indirect/Direct agglutination test: Negative UGT1A1 Gene Polymorphism assay (PCR): TA repeats: 7/7 UGT1A1 genotype: UGT1A1*28/*28			

## 3 DISCUSSION WITH REVIEW OF LITERATURE

### 3.1 Bilirubin metabolism

Bilirubin is the byproduct of heme metabolism. The majority of bilirubin is formed from the breakdown of hemoglobin within senescent RBC, and only around 20% forms from the breakdown of nonheme proteins like cytochrome and myoglobin. Bilirubin so formed is unconjugated and poorly water‐soluble, requiring albumin to circulate in plasma. The bilirubin‐albumin complex thus formed dissociates from albumin as it passes through fenestrated hepatic sinusoidal endothelial cells and reaches hepatocytes. After reaching the hepatocyte, bilirubin is conjugated to bilirubin glucuronide using the enzyme uridine diphosphoglucuronosyl transferase 1. Conjugated bilirubin is secreted across the canalicular membrane of the hepatocyte against a concentration gradient via active transport using canalicular transporters like multidrug resistance protein 2 or ATP‐binding cassette (MRP2/ABCC2).^7^, ^8^ Small proportion of the conjugated bilirubin is secreted back into the sinusoidal blood via the ABCC3 and sinusoidal surface organic anion transporters OATP1B1 and OATP1B3.

**FIGURE 1:**
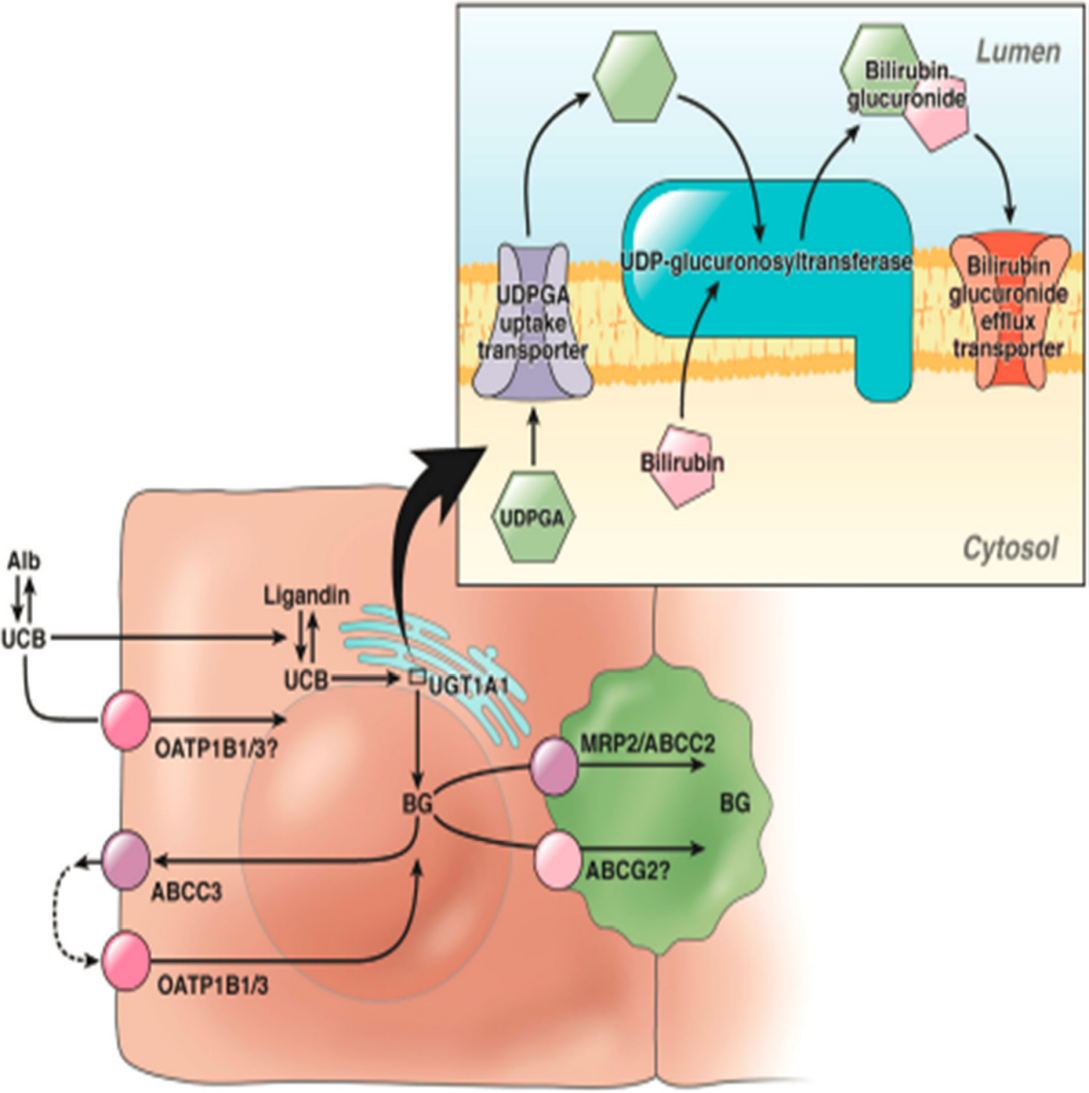
Bilirubin metabolism.^9^

The figure below demonstrates the pathophysiology of bilirubin metabolism as described above (Figure 1):

### 3.2 Familial unconjugated hyperbilirubinemia syndromes

Familial unconjugated hyperbilirubinemia can be categorized into three types based on the activity of the enzyme UDPGT. These types include Gilbert's syndrome, Crigler–Najjar type I, and Crigler–Najjar type II, with enzyme activity reduced by approximately 70%, 100%, and 90%, respectively.^10^

**FIGURE 2:**
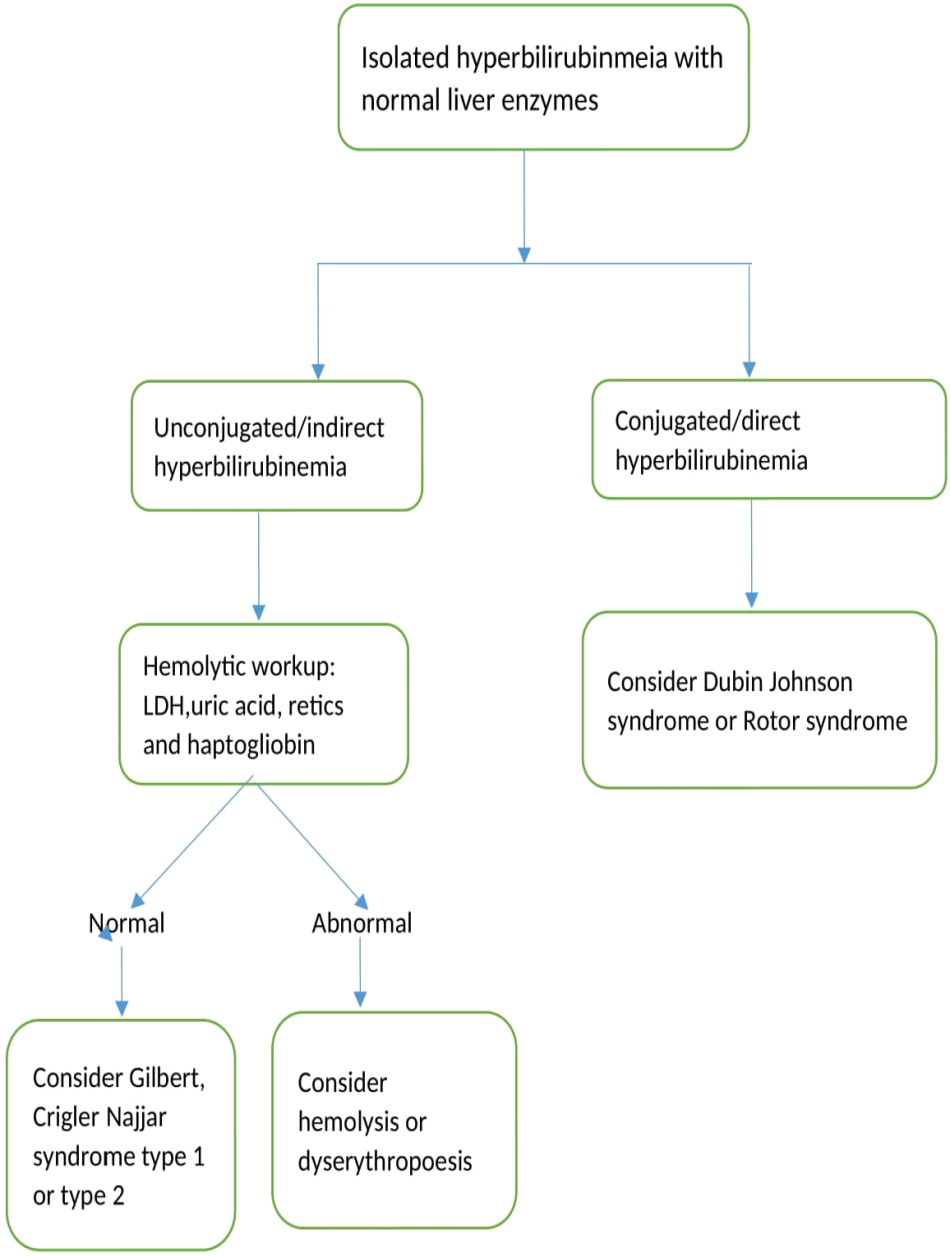
An approach to isolated hyperbilirubinemias.

The algorithm depicted in the flow chart (Figure 2) demonstrates an approach for evaluating the cause of isolated hyperbilirubinemia with a normal liver function test.^11^

#### 3.2.1 Crigler–Najjar syndrome

Crigler–Najjar syndrome (CNS) is a rare genetic disorder with a prevalence of around 0.6–1 case per million live births.^3^ The CNS is characterized by disruptions in bilirubin metabolism, resulting in a persistent elevation of unconjugated bilirubin. The syndrome encompasses two types: type 1 and type 2. The primary underlying cause involves a mutation in the UGTA1 gene, which encodes a liver enzyme called Uridine diphosphate glucuronosyltransferase‐1 (UGT1A1). This enzyme is essential for the conjugation and elimination of bilirubin from the body. In type 1 CNS, there is a complete absence of the enzyme, while in type 2, its presence is partially reduced.^2^ Type 1 represents the more severe form, with newborns rarely surviving beyond infancy due to excessive bilirubin accumulation in the brain. Conversely, type 2 is less severe and carries a more favorable prognosis with patients presenting with features of persistent jaundice exacerbated by stress, intercurrent illness, pregnancy, and drugs.^12^

Crigler–Najjar syndrome should be suspected in a newborn infant or child presenting with features of unconjugated hyperbilirubinemia without evidence of hemolysis or underlying liver disease. In Gilbert syndrome, the level of bilirubin reaches up to 4 mg/dL, and no specific treatment is required for hyperbilirubinemia in this range. In CNS type 1, the concentration of bilirubin is between 20 and 40 mg/dL and the patient requires treatment in the form of either phototherapy, exchange transfusion, or liver transplant. The level of bilirubin in CNS type 2 is less than 20 mg/dL, and treatment can be done medically with phenobarbitone induction therapy.^11^

Phenobarbitone is helpful in the management of type 2 CNS but has no role in the management of type 1 Crigler–Najjar syndrome. Apart from management, phenobarbitone can be a useful diagnostic test for differentiating the type of CNS. The administration of phenobarbital decreases the serum bilirubin by at least 25% for 5 days in type 2, with no or minimal response in type 1.^13^

Complications related to the CNS are because of free, unconjugated bilirubin crossing the blood–brain barrier and binding to a specific part of the brain resulting in necrosis and apoptosis of that part of the brain causing BIND (Bilirubin‐Induced Neurological Dysfunction).^14^, ^15^ It includes a spectrum of neurologic abnormalities, ranging from subtle findings to severe disability.^4^, ^5^ Severe disability, previously called kernicterus, is the extreme form and is associated with permanent neurologic sequelae in the form of gaze palsies, cerebral palsy, and sensorineural hearing loss.^6^ The parts most often affected in the brain include the basal ganglia and the nuclei in the brainstem, which account for oculomotor and auditory function.^16^

A systematic review done by Dhawan et al. has shown that in patients with CNS type 1, unconjugated bilirubin levels increase by 3–6 mg/dL/day during the newborn period and reach neurologically dangerous levels between 5 and 14 days of life. Phototherapy is the mainstay of treatment, and it was found that despite consistent phototherapy, these patients have worsening hyperbilirubinemia with increasing age. Liver transplantation is the only definitive therapy for these patients in terms of survival benefit. But factors like cost, surgical, and medical comorbidities come into play.^17^

#### 3.2.2 Gilbert syndrome

In clinical practice, Gilbert syndrome is the closest differential diagnosis of Crigler–Najjar syndrome type 2 with prevalence ranging between 4% and 16%.^18^, ^19^ Patients with Gilbert syndrome present with features of episodic jaundice often triggered by fever, fasting, hemolysis, or menses occurring typically during the period of adolescence.^20^

No specific treatment is required for patients with gilbert syndrome due to lower level of serum bilirubin contrast to CNS.

The following table helps to differentiate between the three common types of familial unconjugated hyperbilirubinemia (Table 2)^21^:

**TABLE 2:** Comparision of familial unconjugated hyperbilirubinemias.

	Crigler–Najjar syndrome type I	Crigler–Najjar syndrome type II	Gilbert syndrome
Mode of inheritance	Autosomal recessive	Autosomal recessive	Autosomal recessive
Prevalence	Rare	Rare	Common
Prognosis	Poor and progresses to kernicterus if not treated vigorously.	Usually benign	Benign
Serum bilirubin concentration	20–50 mg/dL	Usually 4–20 mg/dL	Usually <4 mg/dL
Liver function tests	Normal	Normal	Normal
Effect of phenobarbitone serum bilirubin	None	Reduction	Reduction
Hepatic bilirubin UGT activity	Absent	Markedly reduced (10% of normal)	Reduced (30% of normal)
Liver histology	Normal, but fibrosis can be present	Normal	Normal

## 4 CONCLUSION

Unconjugated familial hyperbilirubinemia syndromes should be suspected in patients who have presented with isolated indirect hyperbilirubinemia since childhood, in the absence of any apparent cause for the same. These syndromes vary in presentation ranging from fatal consequences in CNS type 1 and benign in CNS type II and Gilbert syndrome. While all these syndromes are related to decreased enzymatic activity of UGT, phenobarbitone is the treatment effective in all except CNS type 1 syndrome. The decision on the assessment of UGT1A1 activity and mutations for the diagnosis should be individualized, and especially in our settings where affordability is an issue, a diagnosis based on the clinical picture remains more important. Our clinical case has been presented to sensitize the clinicians regarding congenital indirect hyperbilirubinemia syndromes, which are themselves rare in occurrence.

## AUTHOR CONTRIBUTIONS

**Divas Rijal:** Conceptualization; methodology; supervision; writing – original draft; writing – review and editing. **Prabhat Rijal:** Conceptualization; methodology; supervision; writing – original draft. **Shyam Murti Bohare:** Conceptualization; writing – original draft. **Ashish Sanjay Chaudhari:** Conceptualization; software. **Mandip Dhungel:** Investigation; methodology. **Mayank Agarwal:** Writing – original draft. **Pramish Bhatta:** Investigation; methodology. **Tulsi Ram Dhakal:** Investigation; methodology. **Anjali Bishwokarma:** Investigation; methodology. **Pooja Kafle:** Conceptualization; resources.

## FUNDING INFORMATION

None.

## CONFLICT OF INTEREST STATEMENT

The author does not possess any conflict of interest.

## CONSENT

Written informed consent was obtained from the patient to publish this report in accordance with the journal's patient consent policy.

## Data Availability

Available from corresponding author on reasonable request.
